# Fabrication of a superhydrophobic porous electrode with voltage-induced anti-fogging properties†

**DOI:** 10.1039/d5ra03009a

**Published:** 2025-05-27

**Authors:** Yaru Ding, Haojie Zhang, Yifan Wang, Jian Li, Yan Zheng

**Affiliations:** a College of Fashion Technology, Zhongyuan University of Technology Zhengzhou 451191 China 6888@zut.edu.cn

## Abstract

The design of stable superhydrophobic surfaces with anti-fogging properties activated by an applied voltage represents one of the most effective methods in the surface and interface sciences. In particular, a flexible three-dimensional (3D) porous electrode capable of resisting high humidity environments (fog) is expected to have a broader range of applications in smart wearable devices. Hence, a superhydrophobic and porous ODT/MWCNTs/MeS electrode has been proposed and investigated for its ability to respond stably under a specific applied voltage. The long alkyl chains of 1-octadecanethiol (ODT) are triggered by the applied voltage to induce chain stretching. The static contact angle of water on the 3D porous electrode is 158.7° at a supply voltage of 0 V, increasing to 162.1° when the voltage is 10 V. The numerous long alkyl chains on the MWCNT coating have been shown to be effective in trapping fog, which can accumulate on the superhydrophobic electrodes to form a liquid film. The *C*–*V* curve of the superhydrophobic electrodes remains almost unaffected at a supply voltage of 10 V, even under continuous fog spray. Moreover, the *i*–*t* curve demonstrates that the continuous fog has minimal impact on the sensitivity and stability of ODT/MWCNTs/MeS electrodes at 10 V. In conclusion, this superhydrophobic and porous electrode, as a smart wearable sensor, can be activated by a supply voltage to achieve a stable response in a humid environment.

## Abstract 2

A superhydrophobic and porous ODT/MWCNTs/MeS electrode has been proposed, and it ability to respond stably with a voltage of 10 V. The long alkyl chains of 1-octadecanethiol (ODT) are triggered by the applied voltage to induce chain stretching.

## Graphical abstract

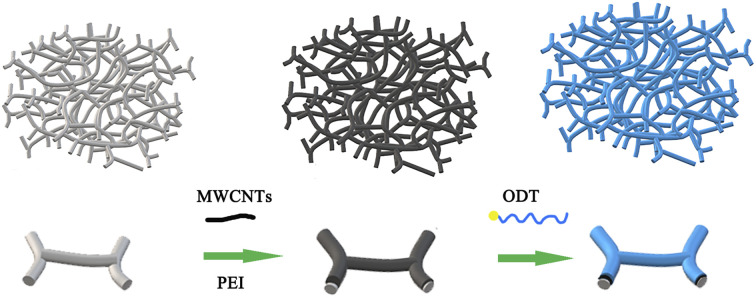

## 1. Introduction

Smart wearable devices play a vital role in the overall health status of an individual, enabling early disease prediction, self-directed diagnostics, personalized therapeutics and improved management of chronic health conditions.^1–3^ However, resisting wetting actions in highly humid environments, including dew, fog, and moisture, is recognized as an effective solution. Water causes wetting of wearable devices, leading to interference and even corrosion of electronic products.^4,5^ Therefore, the superhydrophobic surface of wearable electronics is essential to resist wetting while maintaining high response sensitivity.^6,7^ The air layer of superhydrophobic surfaces has attracted considerable attention because of their superior water isolation capabilities in engineering applications.^8^ However, tiny-droplets can displace air pockets out of the micro/nano rough structure of the superhydrophobic surface, forming a continuous liquid film.^9^ How to achieve a stable air layer on superhydrophobic surfaces and interfaces and prevent the infiltration of tiny droplets is particularly important. Inspired by the leidenfrost droplet,^10^ the liquid drop is levitated above a hot solid surface by a vapor layer generated through evaporation from the drop. Leidenfrost effect can be applied to the superhydrophobic surface of electrodes, where it is expected to maintain a stable non-wetting state.^11^ It is relatively easy to achieve the vapor layer when the superhydrophobic surface is heated by an applied voltage, and this vapor layer is capable of suspending tiny droplets on superhydrophobic surfaces.^12^

However, the effects of temperature on the superhydrophobic surfaces cannot be ignored. At high temperatures, the structure and chemical bonds are destroyed, leading to the loss of superhydrophobicity.^13^ The excellent high-temperature resistance of carbon nanotubes (CNTs) enables them to be used to construct stable roughness in the superhydrophobic surfaces.^14,15^ For example, Zhang *et al.* constructed a durable and photothermal superhydrophobic coating composed of CNTs and silica nanoparticles (SiO_2_).^16^ Low surface energy modification is also critical to achieve a stable superhydrophobic surface for high-temperature conditions. Low surface energy modified materials mainly include PDMS,^17^ silane coupling agents,^18^ long chain alkyls,^19^*etc.* Among these, the chain stretching of long-chain alkyls under electric heating is beneficial for maintaining a stable air layer on superhydrophobic surfaces. Liu *et al.* introduced the hydrophobic 1-octadecanethiol molecular layer, which was functionalized over the Cu catalyst layer of the gas diffusion electrode (GDE), simultaneously stabilizing the gas diffusion electrode (GDL) and exposing abundant active solid–liquid–gas three-phase interfaces.^20^ Carbon nanomaterials consist of carbon atoms held together by strong covalent bonds and exhibit weak chemical affinity and specificity towards other atoms and molecules in its vicinity. Huang *et al.* obtained a graphene/SAM modified Au electrode with the self-assembled monolayer (SAM) of 1-octadecanethiol (C_18_H_37_SH), followed by controllable adsorption of graphene sheets.^21^ Moreover, molecular arrays of long-chain alkyl exhibit stable and durable resistance to tiny droplets. Liu *et al.* developed self-assembled monolayers (SAMs) on Cu substrate to suppress oxidation prior to nano Ag sintering.^22^ This technological approach provides a tangible and cost-effective method for high-temperature electronics packaging. This study demonstrated the influence of 1-octadecanethiol on the hydrophobic coating and the hydrophobic properties of conductive materials. Therefore, the water vapor layer generated by electric heating can replace the air cushion to achieve long-lasting and stable superhydrophobic properties on the porous electronic devices, which can resist the interference of tiny-droplets and fog during mechanical deformation.

Herein, we propose an electrochemically enhanced superhydrophobic property of the 3D porous electrode. The obtained MWCNTs coating is constructed by electrostatic self-assembly to improve electron and phonon transport. ODT formed a superhydrophobic interface on the MWCNTs coating, and ODT/MWCNTs/MeS porous electrode was prepared. Under the action of external voltage, the heat generated by MWCNTs can promote the stretching of long chain alkyl and achieve the stable superhydrophobic surface of electrode to resist the tiny droplets. XPS images show that ODT can adhere to the surface of MWCNTs through π-conjunction, and infrared spectrogram shows the stretching of long alkyl chains under the heat. In the process of electric heating, an obvious vapor layer is formed between the 4 μL droplet and the porous electrode. The fog can also form a liquid film layer on the surface of the electrically heated electrode, and finally, the porous electrode acts as a wearable sensor to generate a stable pressure response signal in a high-humidity environment.

## 2. Experimental section

### 2.1 Materials and methods

Multi-walled carbon nanotubes (MWCNTs, diameter: 10–30 nm, length: 10–300 μm) were purchased from Chengdu Institute of Organic Chemistry. 1-Octadecyl mercaptan (ODT) and polyethyleneimine (MW. 70000, 50% aqueous solution) were purchased from Sigma-Aldrich, China. Ethanol was provided by Fuyu Chemical Co., Ltd. Melamine sponge (MeS) was supplied by Shenzhen Lianda Sponge Products Co., Ltd. Distilled water was commercially obtained. All the solvents were used as received.

### 2.2 Fabrication of MWCNTs/MeS electrodes

A MeS sponge of 1 cm × 1 cm × 2 cm (length × width × height) was cut, and the MeS sponge was washed with acetone and deionized water (DI). Firstly, PEI (100 mg) was dissolved in 100 mL deionized water at room temperature to form a homogeneous solution. Then MWCNTs powder (100 mg) was added to 100 mL anhydrous ethanol, and the mixture was subjected to ultrasonic treatment for 30 minutes to obtain an MWCNTs suspension. The clean sponges were immersed in the PEI solution for 3 minutes and subsequently dried in an oven at 60 °C for 2 hours. After electrostatic modification, the MeS sponge was dipped in the MWCNTs suspension for five minutes and dried in an oven at 60 °C for 2 hours. The self-assembly of MWCNTs was repeated five times to obtain the MWCNTs/MeS electrodes. For the fabrication of the ODT/MWCNTs/MeS electrode, 1-octadecanethiol (6.5 g) was dissolved in 50 mL ethanol at 50 °C to form an ODT solution. Then the MWCNTs/MeS electrode was immersed in the ODT solution for 10 min and dried in a vacuum oven at 70 °C for 8 hours.

### 2.3 Characterizations

Scanning electron microscope (SEM) images were recorded on a Hitachi model S4800. Contact angles were measured with a JCY-1 (Jiangsu Yongrui). X-ray photoelectron spectroscopy (XPS) measurements were conducted on a Thermo ESCALAB 250XI. The surface chemical composition was analyzed by Fourier transform infrared spectrometer (FTIR, ALPHAII). Thermal imaging was captured by infrared cameras. The surface temperature of the electrode was obtained through real-time monitoring by a thermocouple sensor. *C*–*V* curves and *i*–*t* curves were recorded by an electrochemical workstation (Shanghai ChenHua CHI660E).

## 3. Result and discussion

### 3.1 Fabrication of pressure sensor based on porous pyramid structure

**Fig. 1:**
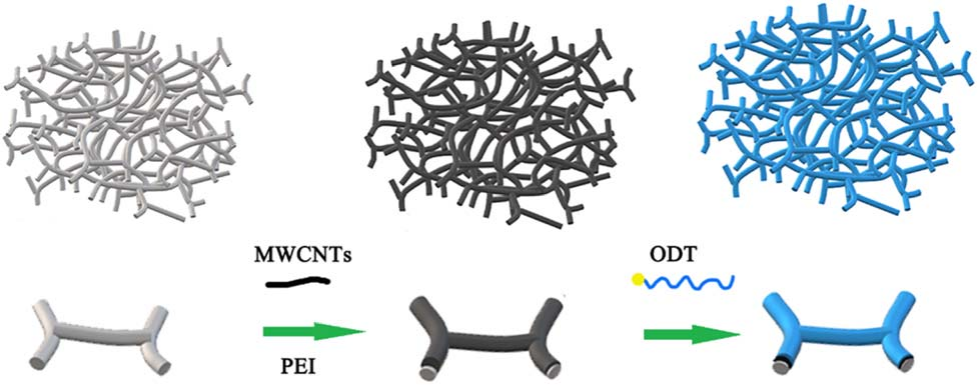
Schematic illustration of the fabrication of ODT/MWCNTs/MeS sponge.

Fig. 1 shows the preparation method of 3D porous electrode. Firstly, the MWCNTs were successfully encapsulated in MeS matrices by the electrostatic self-assembly method, as shown in previous methods.^23^ Subsequently, the ODT coating was self-assembled on the surface of the MWCNTs networks by the dip-coating method. The resulting porous ODT/MWCNTs/MeS electrode exhibited ideal superhydrophobic properties.

**Fig. 2:**
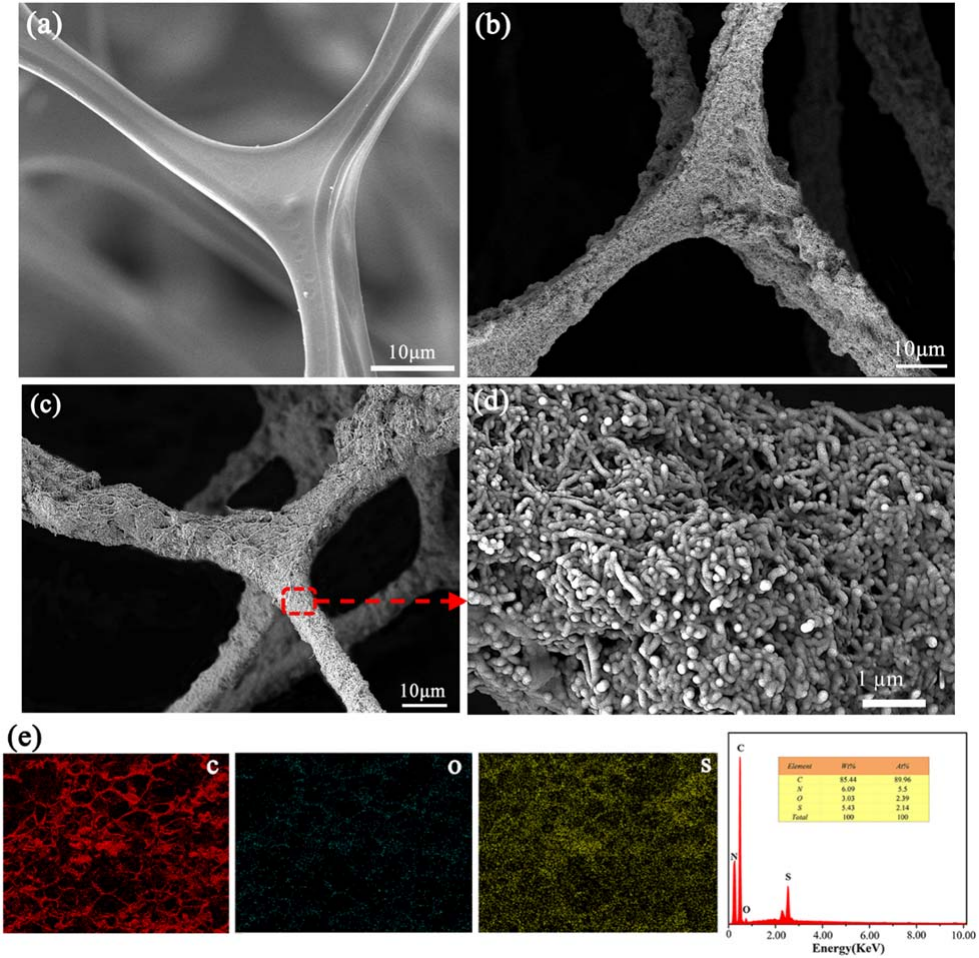
(a) Scanning electron microscopy (SEM) images of MeS sponge, (b) SEM images of MWCNTs/MeS electrode, (c) SEM images of ODT/MWCNTs/MeS electrode, and (d) high magnification images of ODT/MWCNTs/MeS electrode, (e) EDS diagrams of ODT/MWCNTs/MeS electrode.

The surface morphologies of porous electrodes were investigated by scanning electron microscopy. As shown in Fig. 2, it can be clearly seen that the original MeS sponge exhibited an extremely smooth surface at low magnifications, and the holes were about 50 μm in diameter (Fig. 2a). After assembling the MWCNTs coating, the MeS frame has been totally covered, forming a hierarchical micro–nanoscale surface (Fig. 2b). Moreover, an MWCNTs network can be visibly observed from the high magnification image of the MWCNTs/MeS electrode (Fig. S1†). The SEM image of the ODT/MWCNTs/MeS electrode was also analyzed. Compared with MWCNTs/MeS electrode, there is no obvious microstructure change on the surface of ODT/MWCNTs/MeS electrode in Fig. 2c. Furthermore, the magnified view clearly displayed that the bulges of the MWCNTs coating were coated by the self-assembled ODT. The EDS also proved that ODT was uniformly assembled on the surface of MWCNTs, as shown in Fig. 2d. The sulfur (S) elements were uniformly distributed on the MWCNTs coating in Fig. 2e.

**Fig. 3:**
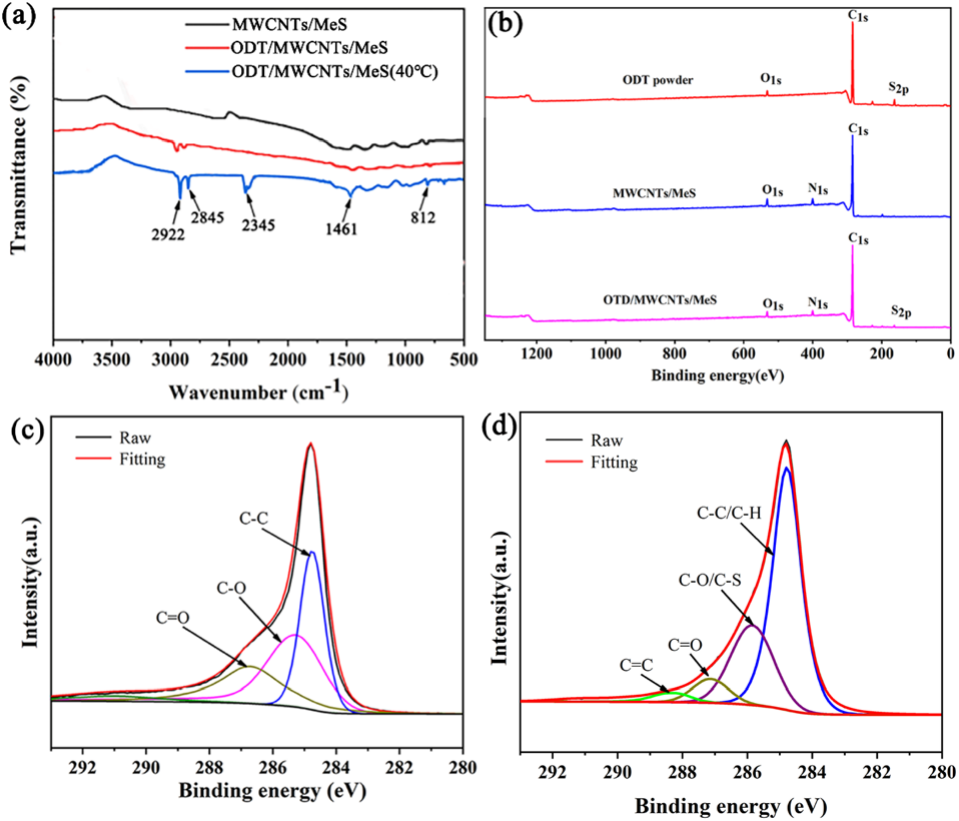
(a) The FTIR spectra of electrodes, (b) the XPS spectra of porous electrodes, (c) wide scanning binding energy of MWCNTs/MeS electrode and (d) ODT/MWCNTs/MeS electrode.

Fig. 3a shows the FTIR spectra of the porous electrode. Loading ODT onto the surface of MWCNTs/MeS electrode, the stretching vibrational peaks at 2922 cm^−1^ and 2845 cm^−1^, corresponds to–CH– and –CH_2_ groups, respectively.^24^ They were from the hydrophobic alkyl chains of ODT. After the ODT/MWCNTs/MeS electrode was heated by ultraviolet light, and its surface temperature reached 40 °C. The strong peak at 1461 cm^−1^ was assigned to C=C sp^2^ from hydrophobic alkyl chains of ODT and MWCNTs. The hydrophobic alkyl chains of ODT and MWCNTs produce strong interfacial interactions and π-conjunction.^25^ Moreover, the absorption peaks of –CH– (2922 cm^−1^) and –CH_2_ (2845 cm^−1^) were significantly enhanced. That favors the elongation of the hydrophobic alkyl chains induced by the high temperature.^26^ The hydrophobic alkyl chains exhibit low intramolecular rotational energy barriers at room temperature. At high temperatures, sufficient energy was provided for the chain segments to overcome these barriers, reducing chain curling and enhancing chain stretching.^27^ Beside, the thiol groups (S–H) of ODT was presented by the sharp C–SH bending peak at 812 cm^−1^ as well as a S–H stretching peak at 2345 cm^−1^, due to the enhanced fluidity of ODT molecular after absorbing heat. Which improves the hydrophobic and anti-fogging properties of the electrode. Fig. 3b shows the XPS general survey spectra of the porous electrodes, revealing a strong S2p signal associated with S–H bonding, confirming the successful assembly of ODT onto the MWCNTs coating. To further illustrate π-conjunction, high-resolution C 1s spectra were analyzed from the MWCNTs/MeS electrode and the ODT/MWCNTs/MeS electrode. Compared to MWCNTs/MeS electrode (Fig. 3c), it discovered that the peaks of C–S bonding at 286.4 eV and C–C bonding at 284.8 eV on the ODT/MWCNTs/MeS electrode. As shown in Fig. 3c, the peaks of C=C bonding at 288.6 eV, which comes from the π-conjunction between the long chain alkyl of ODT and MWCNTs. The XPS results conclusively demonstrate the successful functionalization of the MWCNTs coating with hydrophobic ODT molecules.

**Fig. 4:**
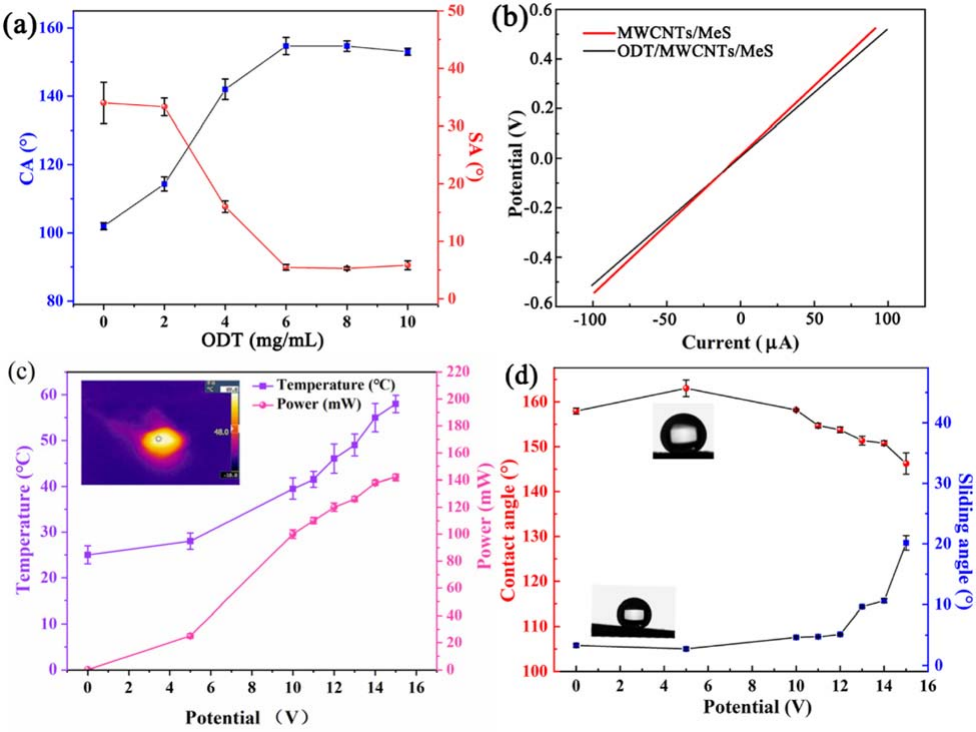
(a) The water contact angle (CA) and (sliding angle) SA of ODT/MWCNTs/MeS electrodes. (b) Electrical property of porous electrode. (c) Temperature and power dissipation as the increasing of supply voltage. (d) The water CA and SA of ODT/MWCNTs/MeS electrodes as increasing of the voltage.

The wettability of ODT/MWCNTs/MeS electrode was studied using deionized water (DI) of 8 μL. At an ODT concentration of 6 mg mL^−1^, the electrode exhibited superhydrophobic properties, with a high water contact angle (CA) of 154 ± 1° and a low slip angle (SA) of 5.2 ± 0.1°, as illustrated in Fig. 4a. In addition, the *C*–*V* curves of ODT/MWCNTs/MeS electrode and MWCNTs/MeS electrode show similar electrical properties, and Fig. 4b shows that the ODT coating has almost negligible effect on the electrical properties of the MWCNTs/MeS electrode. However, heat is generated during ODT/MWCNTs/MeS electrode operation, and it is necessary to explore the temperature, power dissipation, and wettability changes on the ODT/MWCNTs/MeS electrode. As shown in Fig. 4c, the temperature of the electrode is recorded by a thermocouple sensor. When the voltage exceeds 5 V, the temperature rapidly increases to 28 °C on the ODT/MWCNTs/MeS electrode. The power loss of the electrode is 20 mW. While the power loss is calculated by formula (1).

1 *P* = *UI*

Here, *P* denotes the power loss of the electrode, *U* represent the supply voltage of the electrode, *I* is the current passing through the electrode. The power loss (*P*) of the ODT/MWCNTs/MeS electrode is converted to Joule heat. An increasing of power loss will lead to higher surface temperature of electrode. Under the supply voltage of 10 V, the surface temperature of electrode at 38 °C, and the power loss of the electrode is 100 mW. The water CA decreases to 158 ± 1°, and the SA increases to 5.2 ± 0.1°, which still shows a superhydrophobic ODT/MWCNTs/MeS electrode (Fig. 4d). However, as the increasing of supply voltage, the electrode transitions to a pinning superhydrophobic surface. Therefore, excessive supply voltage may potentially damage the hydrophobic performance of the electrode.

**Fig. 5:**
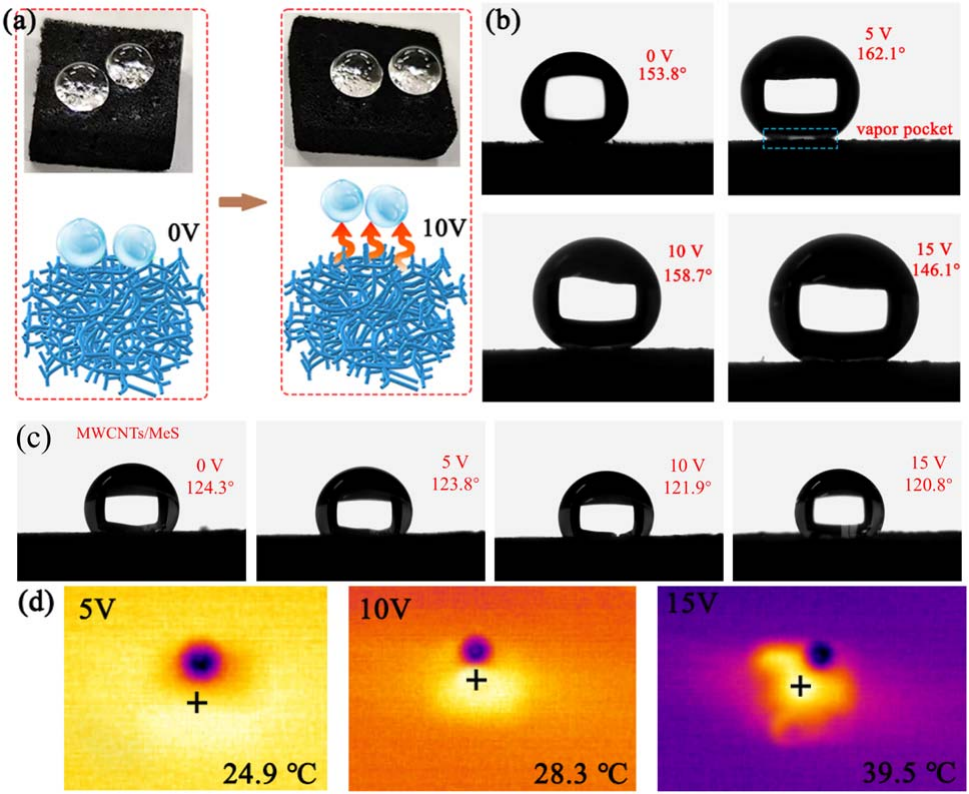
The CA of ODT/MWCNTs/MeS electrodes under applied voltage. (a) The water droplet state on the electrode surface. (b) The CA of 158.7° with 0 V, 162.1° with 5 V, 153.8° with 10 V, 146.1° with 15 V. (c) The CA of MWCNTs/Mes electrode with 0 V, 5 V, 10 V, 15 V, respectively. (d) The thermal imaging of a droplet on the electrode surface under different supply voltages.

The higher supply voltage increases the surface temperature of electrode, triggering the stretching vibration of long alkyl chains in ODT molecules. The long alkyl chains can reduce the surface energy and enhance the superhydrophobic property of ODT/MWCNTs/MeS electrode. Fig. 5 provides the morphology of water droplets (4 μL) on the surface of the ODT/MWCNTs/MeS electrode as the supply voltage increases. Fig. 5a shows the spherical water droplet on the ODT/MWCNTs/MeS electrode with a CA of 158.7 ± 1° at the supply voltage of 0 V, and a slight contact overlap is generated at the water droplet/electrode interface. When the voltage increases to 5 V, the water droplet remains spherical, and a vapor pocket forms at the water droplets/electrode interface with a CA of 162.1°. Meanwhile, the surface temperature of electrode at 28 °C, the alkyl chains stretching of ODT and the hydrophobic properties are enhanced. However, when the voltage increases to 10 V, the vapor pocket weakens at the water droplet/electrode interface and the water droplet remains sphere. Due to the surface temperature of electrode at 38 °C, which causes the ODT to melt, resulting a decrease of hydrophobic performance. When the voltage is increased to 15 V, the vapor pocket disappears on the electrode surface. The CA of electrode decreases to 146.1 ± 1°, and the electrode transitions to a hydrophobic surface. The surface temperature of electrode reached to 56.8 °C under 15 V supply voltage. Meanwhile, Fig. 4d shows that the CA of the electrode is 146.1 ± 1°, and a 10°of sliding Angle (SA). These show the deterioration of the hydrophobic surface. The liquefied ODT is lost from the MWCNTs coating, and deteriorated the hydrophobic surface. As a control test, the CA of MWCNTs/MeS electrodes is used in the same conditions in Fig. 5c. The CA of electrodes was 124.3° without power supply. As the supply voltage increasing, the CA of electrodes decreased, showing a trend of enhanced hydrophilicity. Thus, on the raw MWCNTs/MeS electrodes, even if the voltage is increased to raise a surface temperature of electrode, the surhydrophobic effect on MWCNTs coating cannot be achieved. Fig. 5d shows the heat absorption of 2 μL droplets (blue dots) on the ODT/MWCNTs/MeS electrode surface. The droplet shows a relatively low temperature with blue color, and there is an orange heat-absorbing area on the outer layer of the droplet. These proves that a vapor pocket at the droplet/electrode interface. Therefore, the ODT coating on the MWCNTs/MeS electrodes was important to achieve the Leidenfrost-like effect.

**Fig. 6:**
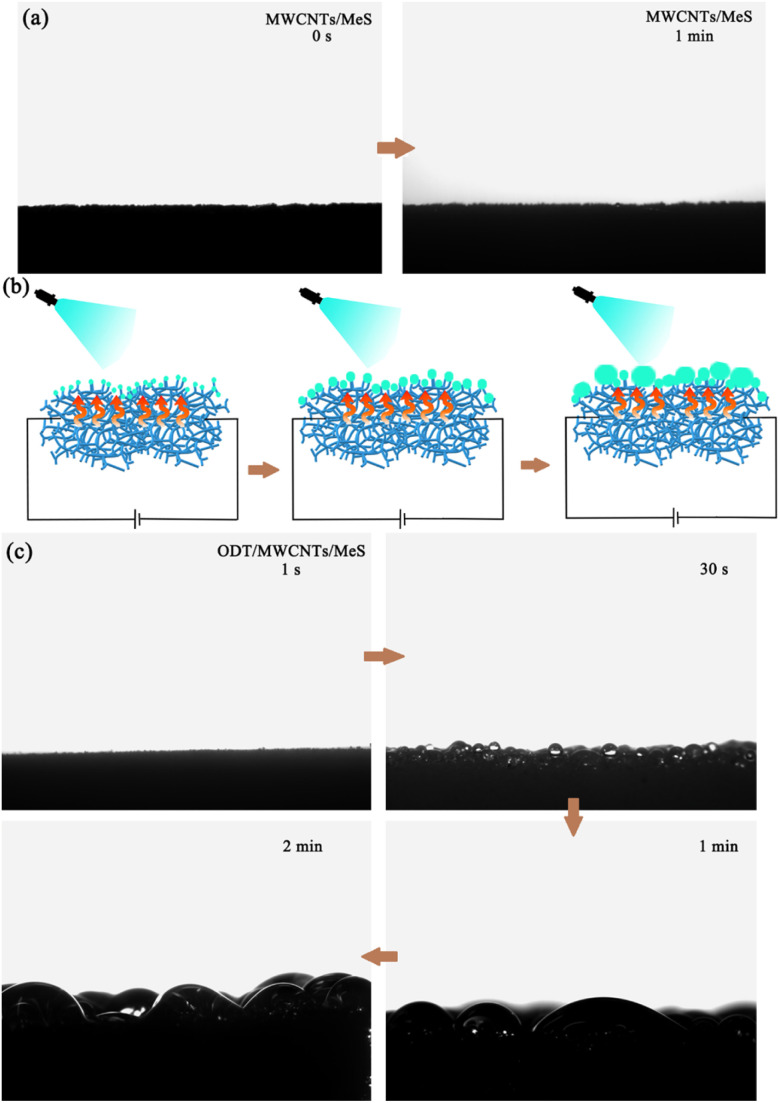
Condensation of fogs on the electrode, spraying MWCNTs/MeS electrodes lasts 0 s (a) and 1 minute with fog, (b) formation of condensed droplets on superhydrophobic surface, (c) spraying ODT/MWCNTs/MeS electrodes lasts 1 s, 30 s, 1 minute, 2 minutes.

The dynamic condensation of the porous ODT/MWCNTs/MeS electrode on a superhydrophobic surface was studied. When the supply voltage reaches 8 V, the nucleation and coalescence of fog droplets on the superhydrophobic surface are illustrated in Fig. 6 and Movie S1.† As shown in Fig. 6a, after 1 minute, the wetting of fog droplets into the MWCNTs/MeS electrode occurs, with almost no droplets remaining on the surface of the electrode. Even with the MWCNTs coating, the fog droplets could quickly penetrate the MWCNTs/MeS electrode, forming a water film. The condensation process can be divided into three stages: (1) the initial growth of small droplets, (2) an immobile state, and (3) the formation of a liquid film under electric heating, as shown in Fig. 6b. In the first stage (Fig. 6c), fog nucleation occurred in the initial stage on the superhydrophobic frame, and the condensed droplets distributed randomly on the superhydrophobic frames. In stage 2, droplets on the superhydrophobic frames coalesced with each other in the vicinity, forming lager stationary droplets. On an electrically heated superhydrophobic surface, the coalesced droplets merged on the porous electrode. In stage 3, the condensed droplet continued to coalesce and, forming a liquid film during the fog spraying process, demonstrating the potential applications of the superhydrophobic surface in wearable devices.

**Fig. 7:**
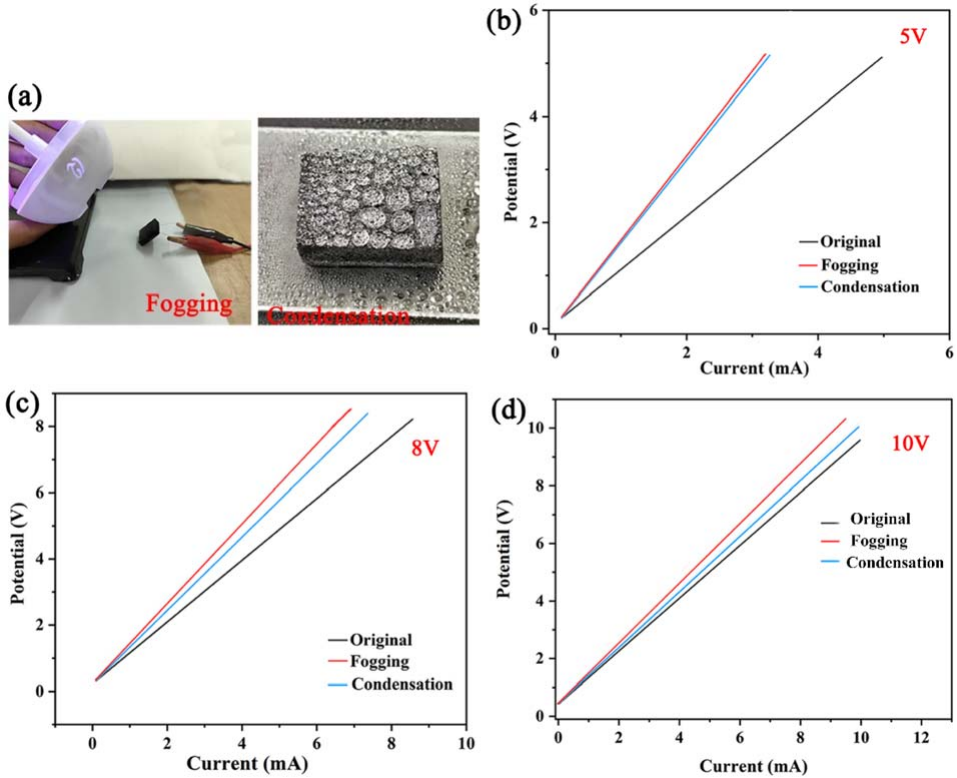
(a) The photographs of original ODT/MWCNTs/MeS electrodes, fog humidification and condensation on the surface of electrodes. The *C*–*V* curves of ODT/MWCNTs/MeS electrodes at 5 V (b), 8 V (c), 10 V (d) external voltage and three wetting states.

Fig. 7 displays the electrical properties of the ODT/MWCNTs/MeS electrodes under different supply voltages. As shown in Fig. 7a, the ODT/MWCNTs/MeS electrode is connected to a two-electrode system, with a humidifier used to generate fogging. The *C*–*V* curves of the electrode under three conditions-original, fogging, and condensation are recorded. When the voltage is set to 5 V, the *C*–*V* curve of ODT/MWCNTs/MeS electrode differs from the original after fogging and condensation, which indicates that the fogging interferes with the electrochemical performance of the electrode (Fig. 7b). When the voltage is 8 V, the difference in the *C*–*V* curves for fogging, condensation, and original electrode gradually decreases (Fig. 7c). At a supply voltage of 10 V, the *C*–*V* curve of the condensation electrode is similar to that of the original electrode, proving that the resistance of electrode to fogging interference gradually improves as the operating voltage increases (Fig. 7d).

### 3.2 Working principle and performance of pressure sensor

**Fig. 8:**
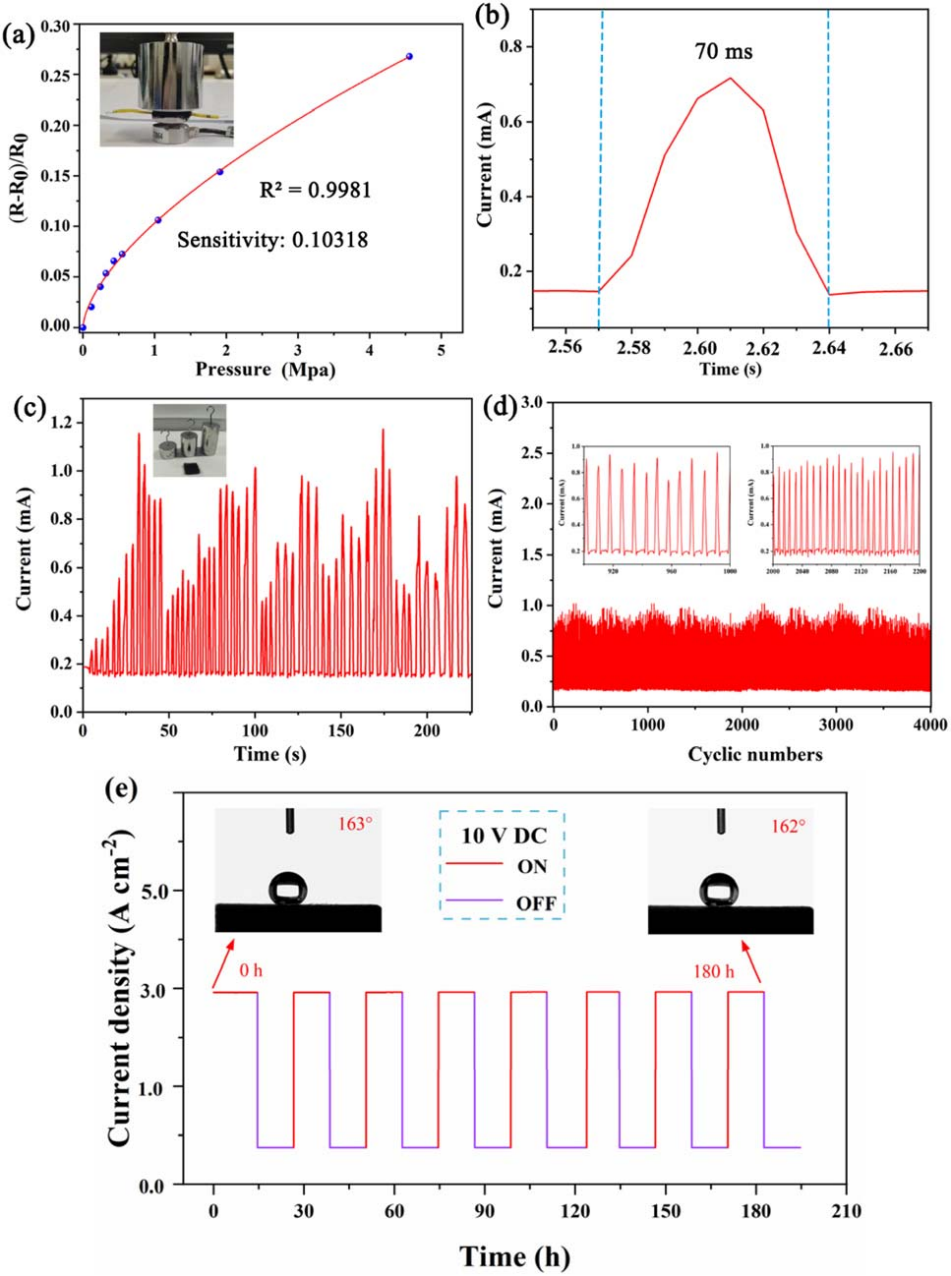
The sensing performance of superhydrophobic pressure sensor based on porous ODT/MWCNTs/MeS electrode, (a) the relative resistance change of pressure sensor based on ODT/MWCNTs/MeS electrode, (b) the responsive and recovery time for the strain sensor under the strain 5%, (c) the cyclic dynamic response for the strain sensor under different strains of 10–87%, (d) dynamic cycling performance for 4000 cycling tests under cyclic tensile loading and unloading of 25%, and the magnified view. (e) Current response and hydrophobicity of ODT/MWCNTs/MeS electrode for long time 10 V DC On/Off cycles.

The ODT/MWCNTs/MeS electrode is used as a resistance-type pressure sensor. Under applied pressure, the thickness of the porous electrode is decreased, and the electric conduction is increased as shown in Fig. 8a. Sensitivity (*S*) is defined as:

2 *S* = (Δ*R*/*R*_0_)/*P*

where Δ*R* is the change in resistance, *R*_0_ is the initial resistance, and *P* is the applied pressure. The sensitivity is inversely proportional to the compressive modulus of the elastomer (*i.e.*, a larger change in the diameter of hole at a given pressure yields higher sensitivity). The porous structure increases the sensitivity firstly by drastically decreasing the effective compressive modulus of the elastomer. Moreover, Fig. 8b shows a response and recovery time of 70 ms, exhibiting the fast response performance for wearable pressure sensing device. As shown in Fig. 8c, the strain range gradually increases from 10% to 87% at a supply voltage of 1 V, with corresponding resistance producing a step change. Fig. 8d displays the current response curve of pressure sensor under 4000 repeated loading–unloading cycles of pressure at 400 kPa, showing high repeatability and durability. To evaluate the practicality of ODT/MWCNTs/MeS electrode, the electrode maintained alternating 15 hours charging and 15-hours discharging under a 10 V DC power supply for 180 hours. As shown in Fig. 8e, compared with the initial ODT/MWCNTs/MeS electrode, the change of supply voltage does not affect the electrical response stability. After power-on and power-off cycling tests confirmed that the ODT/MWCNTs/MeS electrodes did not undergo oxidation to decrease conductivity. In addition, the stable superhydrophobicity confirmed that the ODTs did not desorb or pyrolyze under the 10 V supply voltage.

**Fig. 9:**
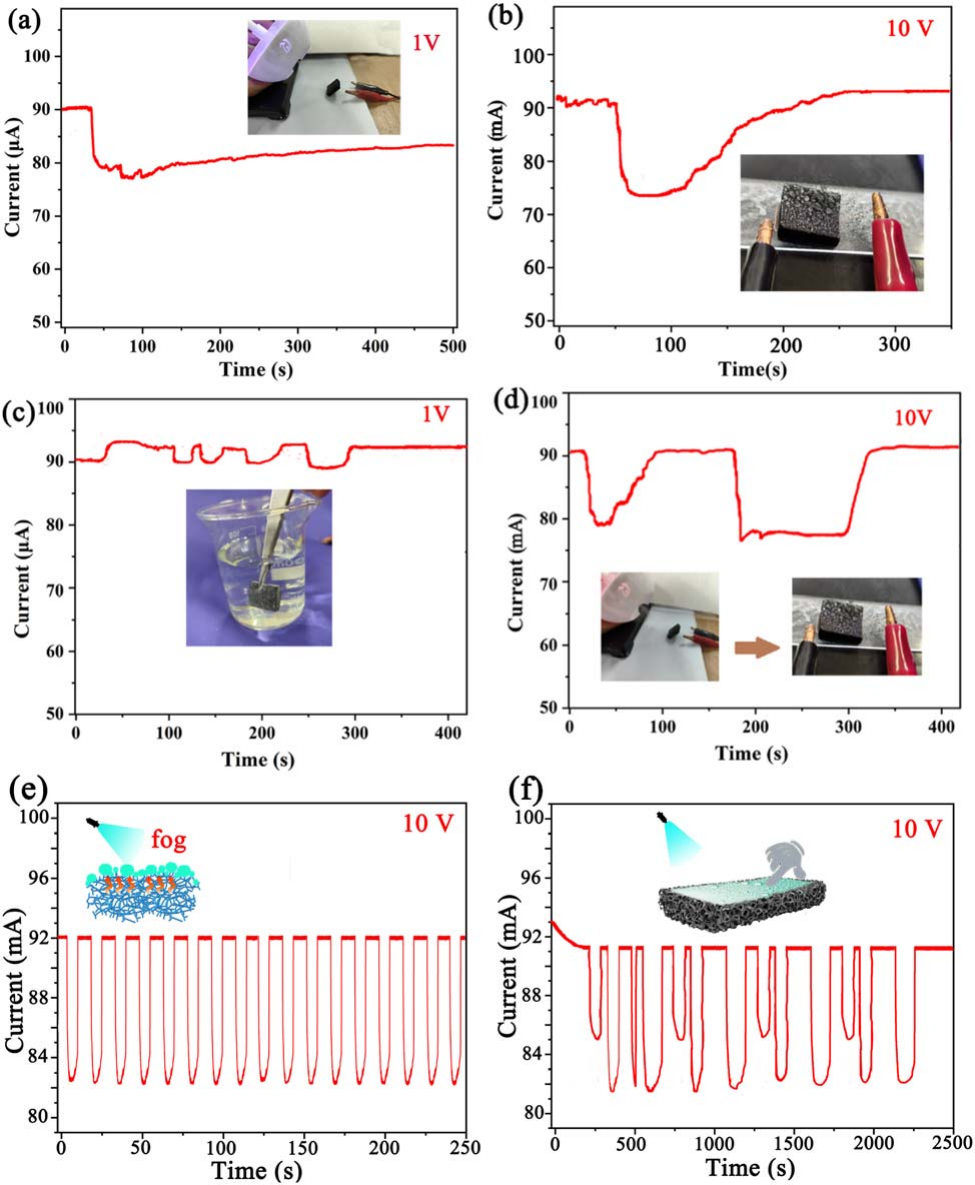
(a) The fogs condense on the surface of ODT/MWCNTs/MeS porous electrode. (b) After the droplets condense on the ODT/MWCNTs/MeS electrode, and the electrode is heated with a hair dryer. (c) The electrodes are soaked in hot water. (d) The fogs condense on the ODT/MWCNTs/MeS porous electrode with an external voltage of 10 V. (e) The fogs are intermittently sprayed onto the ODT/MWCNTs/MeS electrode at 10 V. (f) Pressing the ODT/MWCNTs/MeS electrode with a water film.

To determine the response of the ODT/MWCNTs/MeS electrode under electric heating, the *i*–*t* curve of electrode was further recorded. As shown in Fig. 9a, when an external voltage of 1 V is applied, the current of the porous electrode decreases after fog spraying. With the continuous fogging, the current exhibits a certain rebound, but does not fully return to its original state. Fig. 9b shows that after spraying, the current of the electrode quickly recovers to its initial state at a supply voltage of 10 V. Moreover, to examine temperature response of the ODT/MWCNTs/MeS electrode, the electrode was repeatedly soaked in hot water at 40 °C. As shown in Fig. 9c, the current increases upon immersion in hot water and returns to its original state once removed. Finally, Fig. 9d shows that the fog spraying reduces the current of the electrode with the supply voltage of 10 V. However, the current quickly recovers after fogging stops, even in the presence of a condensation layer on the electrode surface. To further analyze the response stability of the ODT/MWCNTs/MeS electrode under fog spraying. An intermittent spraying method was adopted to test the response of superhydrophobic electrode at a supply voltage of 10 V in the Fig. 9e. When the fogs are sprayed onto the electrode surface, the current of electrode decreases. After the spray is stopped and the condensed water is absorbed dry, the current of electrode can return to its initial state. Notably, the continuous spraying of fogs will form a liquid film on the electrode surface. After the liquid film is formed, the electrical response of the electrode will no longer be affected by the fogs. Pressing the electrode with a finger will generate a pressure response in the Fig. 9f. Thus, under a supply voltage of 10 V, the electrode continuously generates heat, which can prevent fogs from entering the interior of the electrode and thereby maintain the stability of the electrode. However, when a condensation water film accumulates on the electrode surface, the fogs will no longer directly act on the electrode surface. The response performance generated by pressure stimulation will not be affected by the fogs.

## 4. Conclusion

Piezoresistive smart sensors have experienced rapid growth due to their flexibility, breathability, and ease of use in sensing human motion and measuring pressure. In this study, the ODT/MWCNTs/MeS electrode was successfully fabricated as a wearable sensor with stable superhydrophobic properties. Infrared spectroscopy results showed that the stretching of long-chain alkyl groups in ODT contributed to maintaining a stable superhydrophobic surface at 40 °C. Under a supply voltage of 5 V, a 4 μL droplet was able to maintain on the electrode surface, achieving a contact angle of 162.1°, with a vapor pocket forming between the droplet and the electrode. Moreover, even when fog was sprayed onto the electrode surface at 10 V, the electrical performance remained almost unaffected. When used as a sensor, regulating the supply voltage of the electrode can result in a stable pressure response signal in high humidity environments. Overall, this study highlights the advantages of integrating electrical heating with superhydrophobic surfaces and demonstrates significant potential applications in smart wearable electronics.

## Data availability

All data generated or analyzed during this study are included in this article.

## Author contributions

Yaru Ding: acquisition of fundings, methodology, data analysis, review of manuscript. Haojie Zhang: performed experiments, collected data, prepared manuscript. Yifan Wang: hydrophobicity analysis and edited manuscript. Jian Li: mechanical bending analysis and reviewed manuscript. Yan Zheng: project management, reviewed manuscript.

## Conflicts of interest

The authors declare that they have no competing financial interests or personal relationships that could influence the work reported in this study.

## Supplementary Material

RA-015-D5RA03009A-s001
