# Supplementary material for: Fabrication of a superhydrophobic porous electrode with voltage-induced anti-fogging properties
Source: RSC Adv. 2025 May 27;15(22):17435–41. doi: 10.1039/d5ra03009a (PMC12107341; doi:10.1039/d5ra03009a)
Supplement: RA-015-D5RA03009A-s001 [file RA-015-D5RA03009A-s001.pdf]

Supplementary Information (SI) for RSC Advances.
This journal is © The Royal Society of Chemistry 2025

Supporting Information

Fabrication of a Superhydrophobic Porous Electrode with Voltage-Induced Anti-Fogging Properties

Yaru Ding [a] (✉), Haojie Zhang [a], Yifan Wang [a], Jian Li [a], Yan Zheng [a]

Yaru Ding [a] (✉), e-mail: 6888@zut.edu.cn.

a: College of Fashion Technology, Zhongyuan University of Technology, Zhengzhou 451191, China.

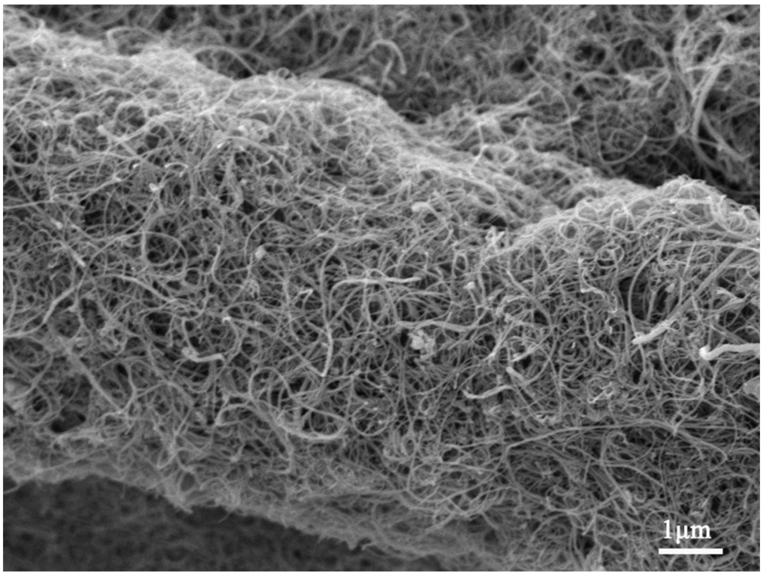

Fig. S1 image of the MWCNTs/MeS electrode
